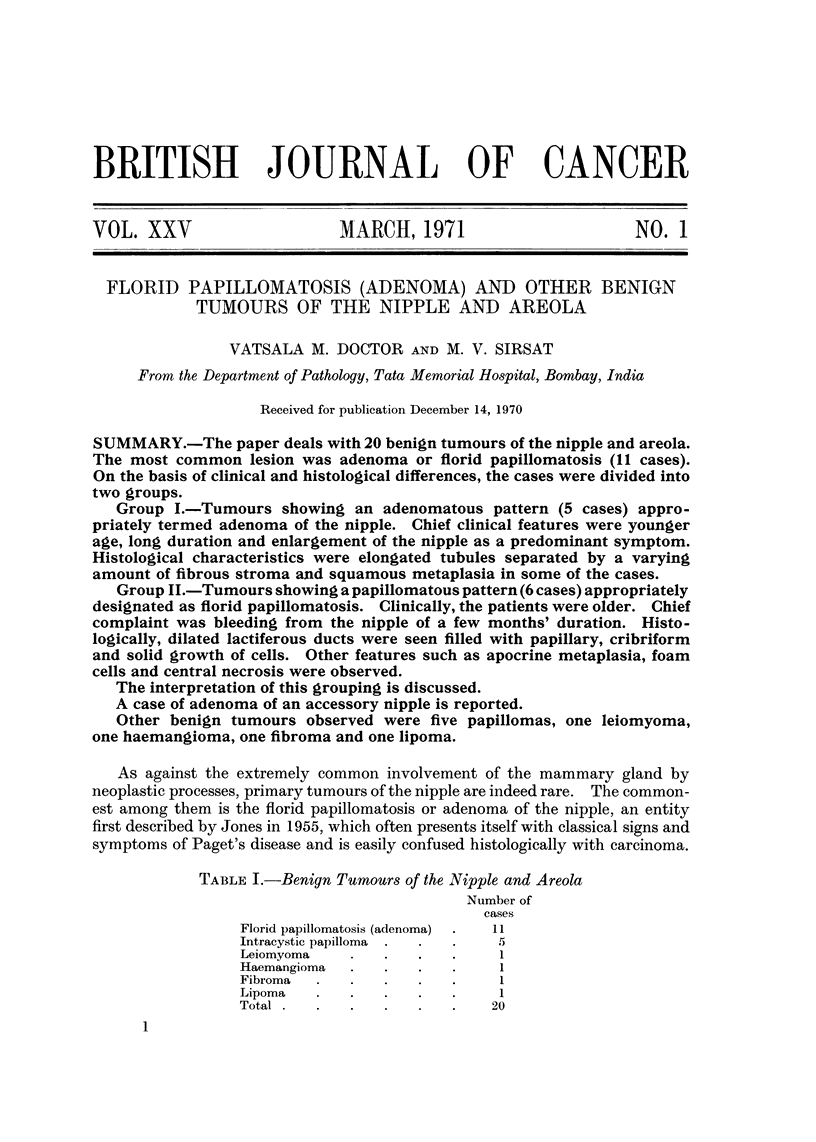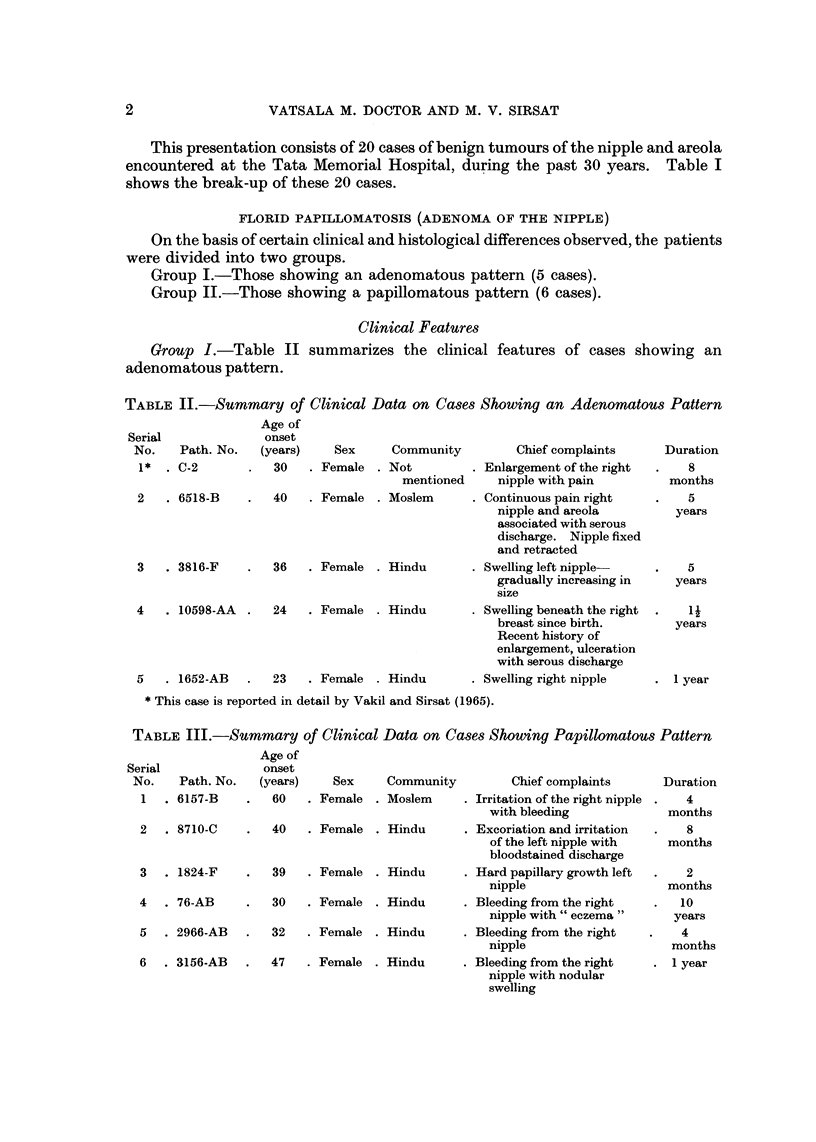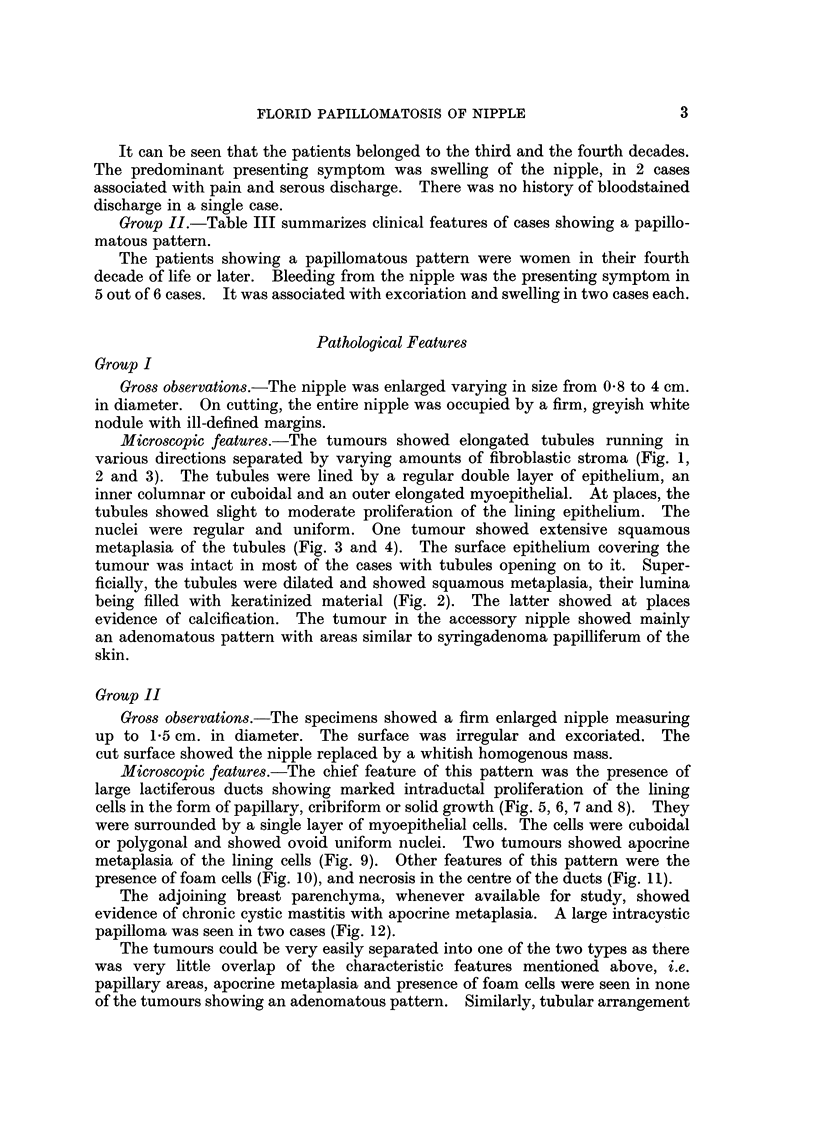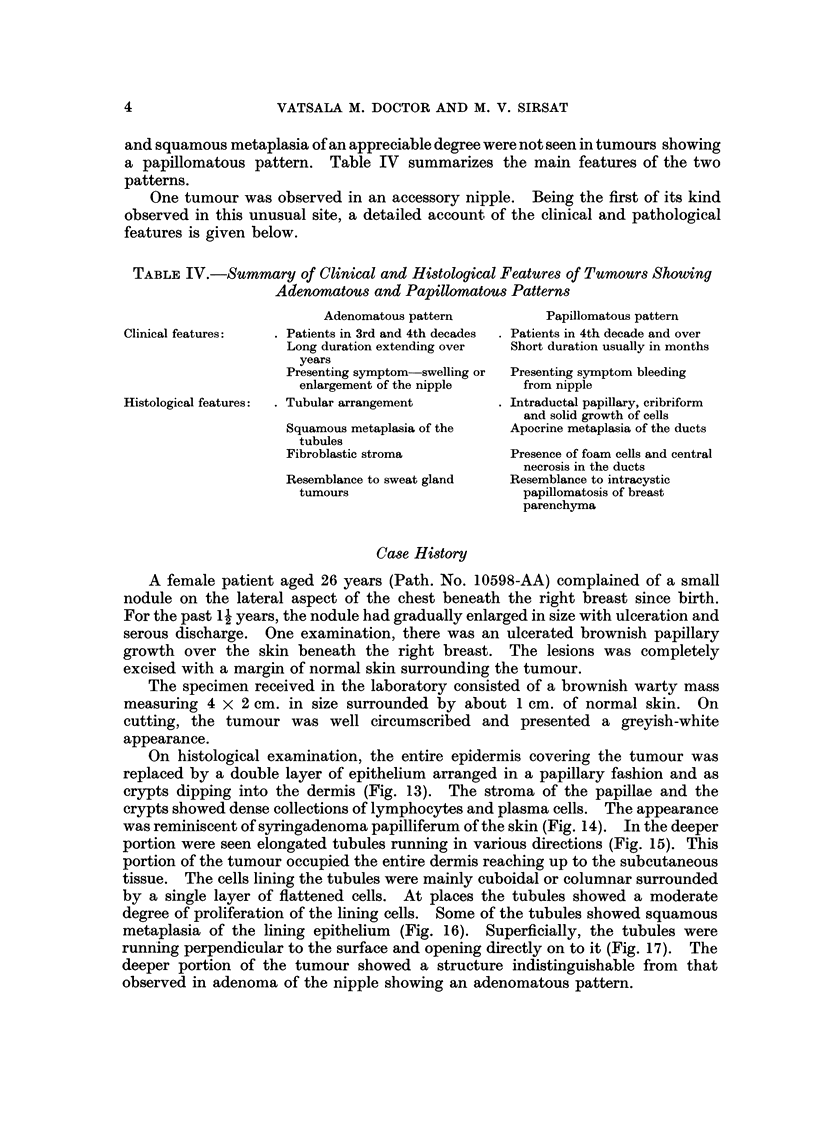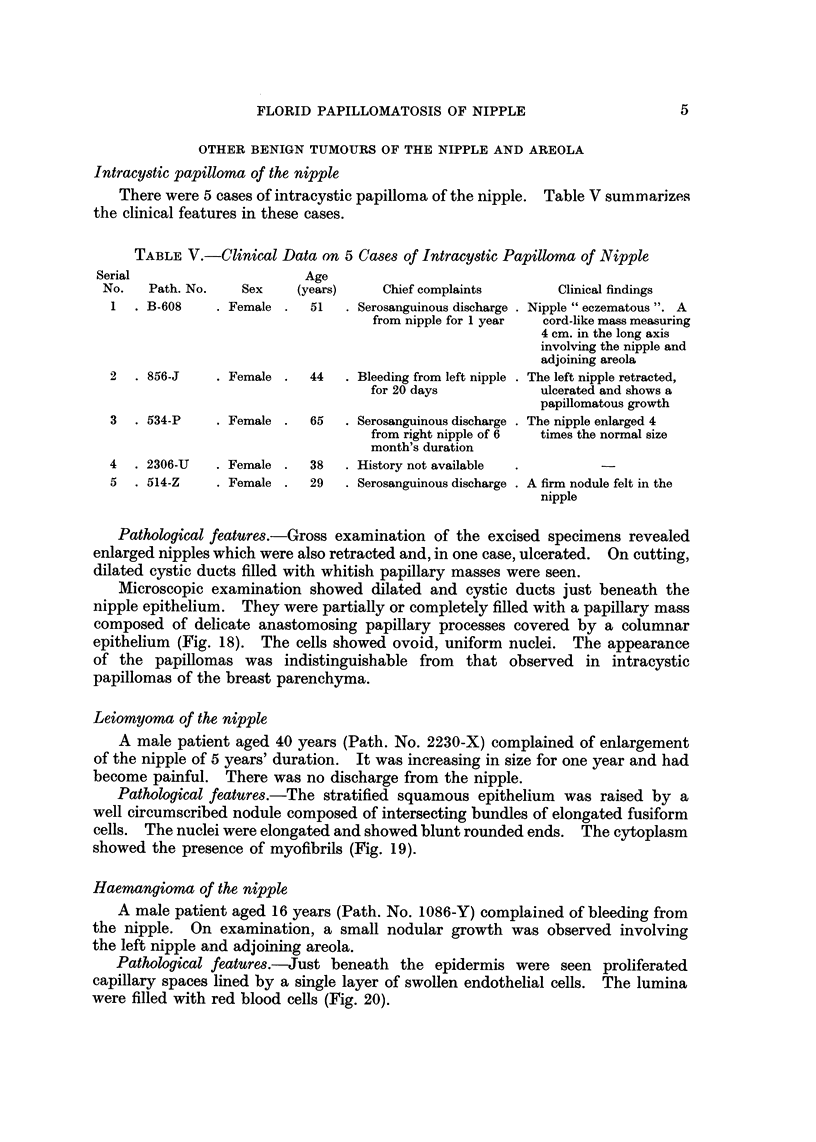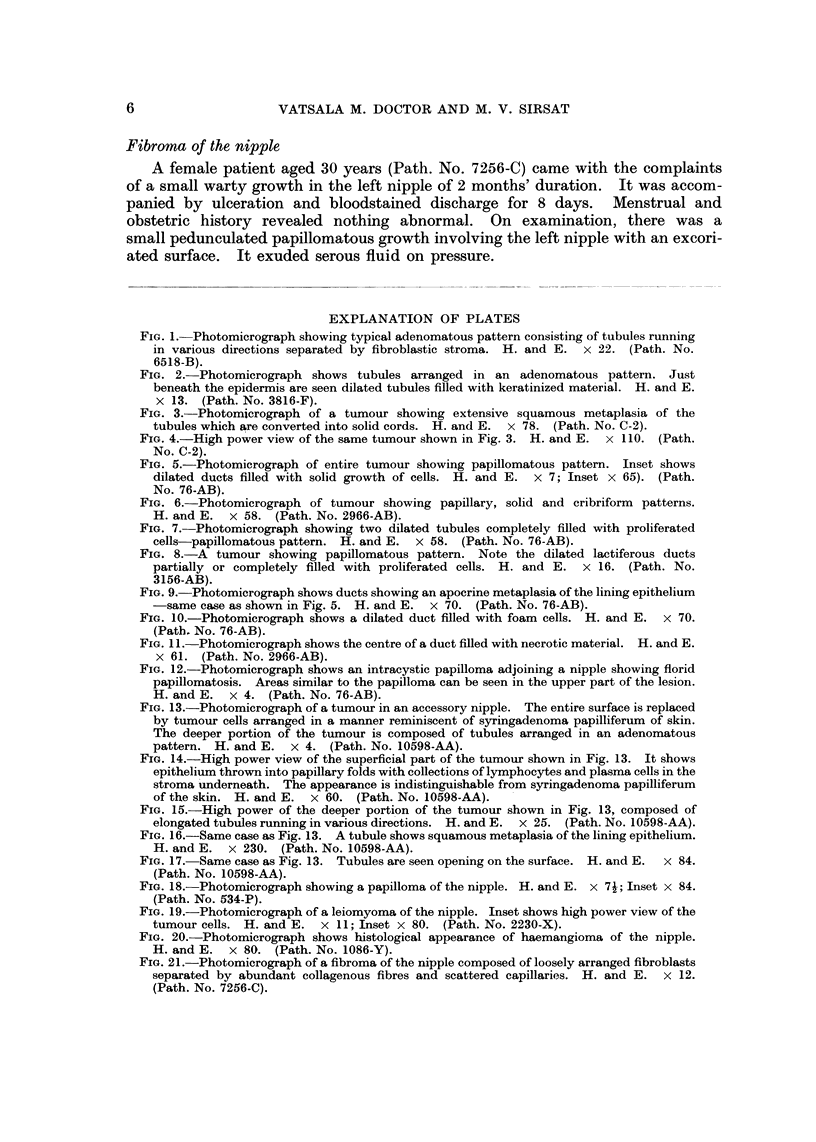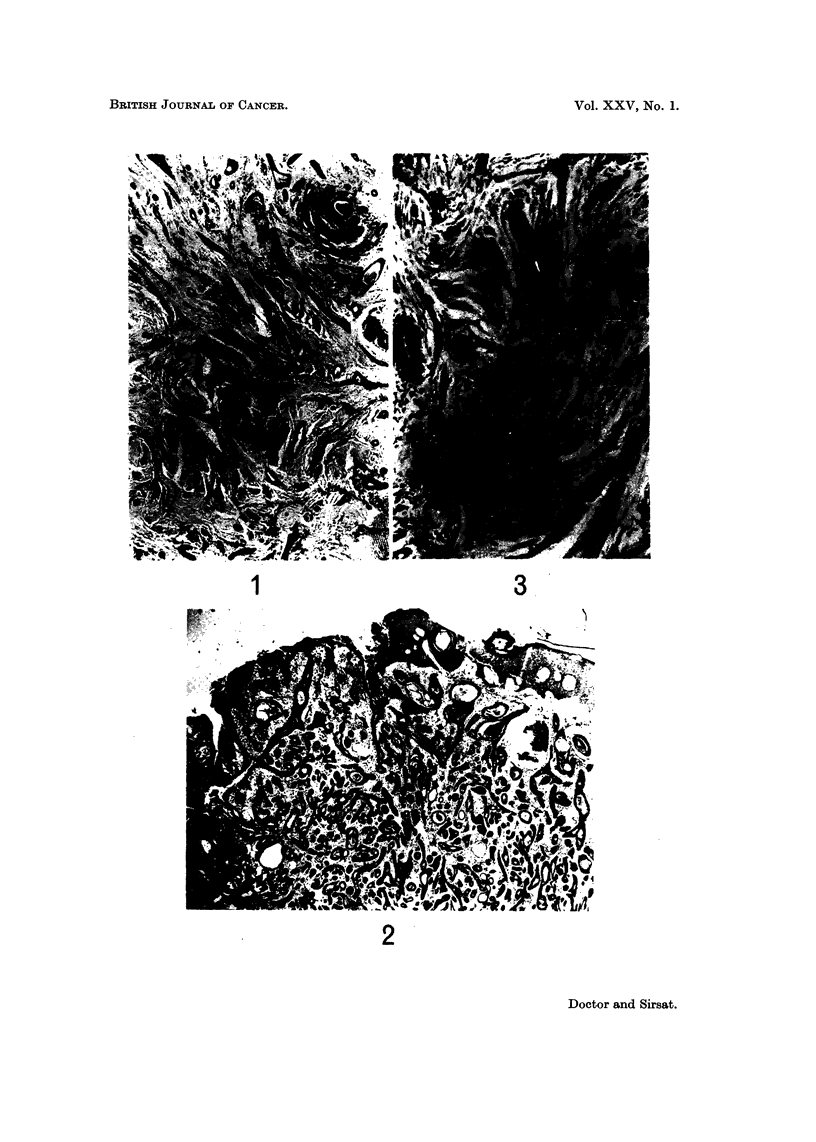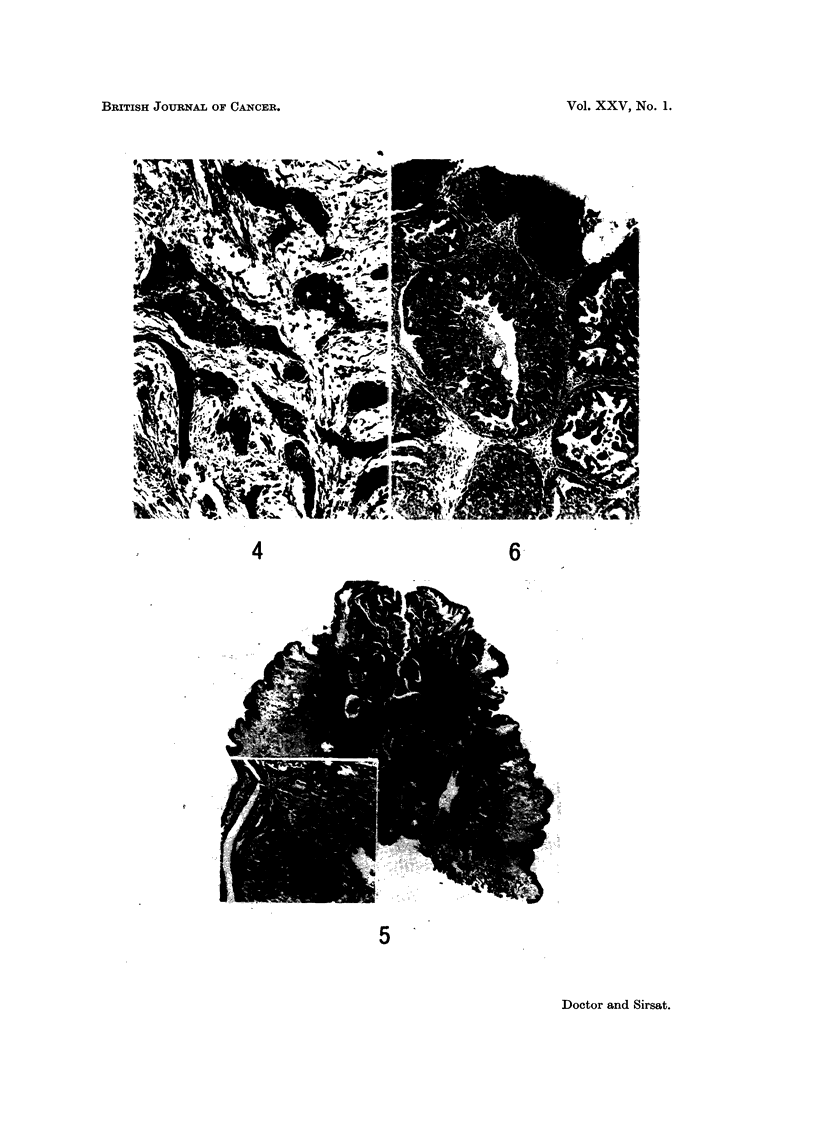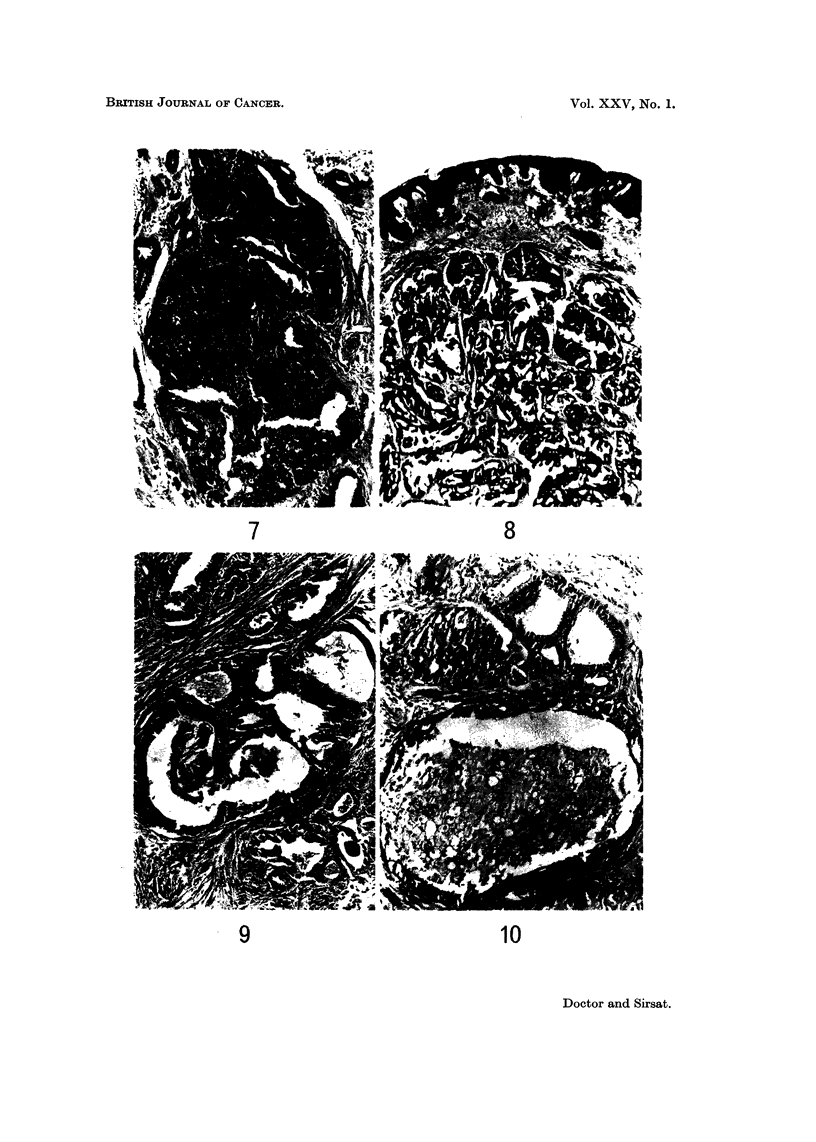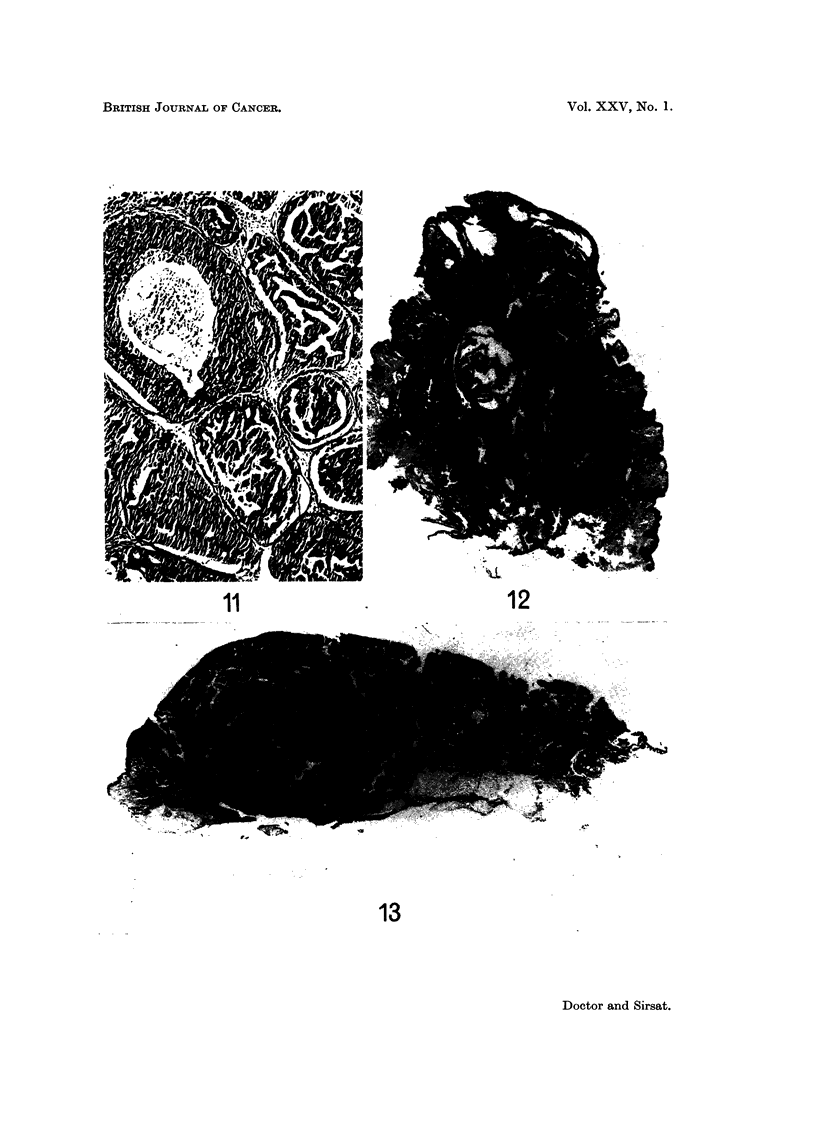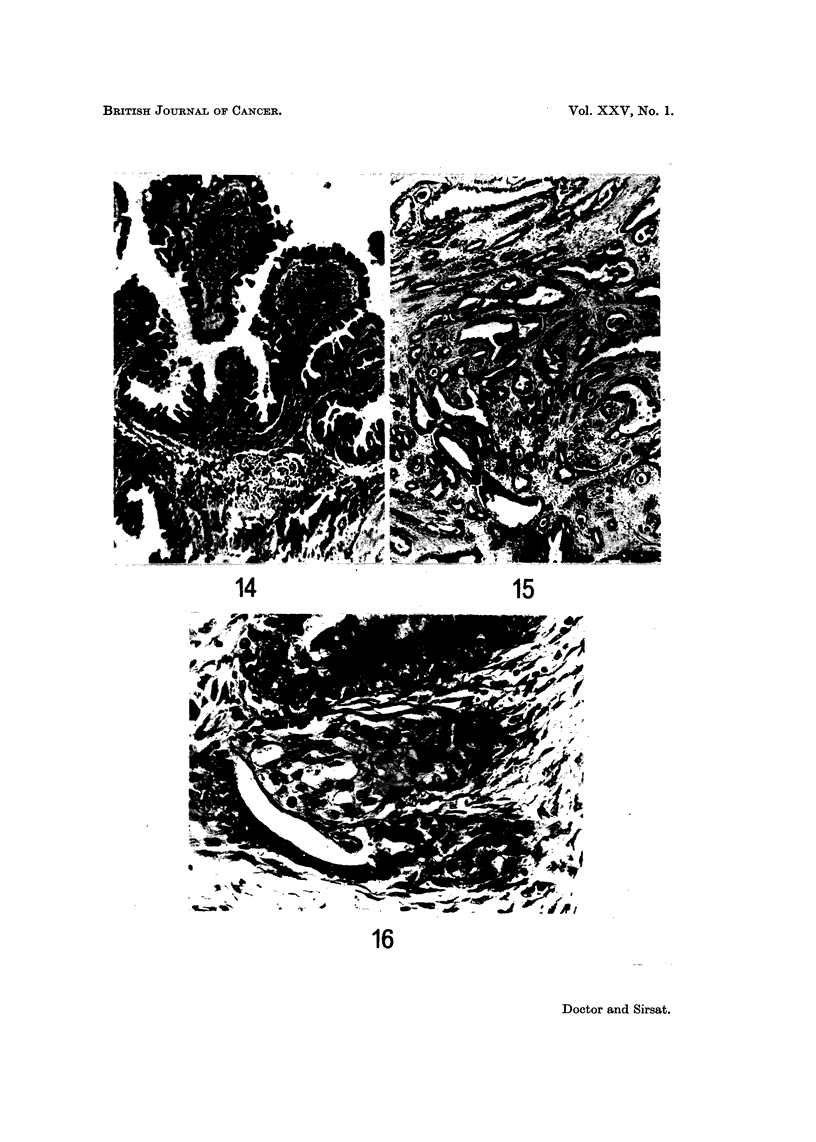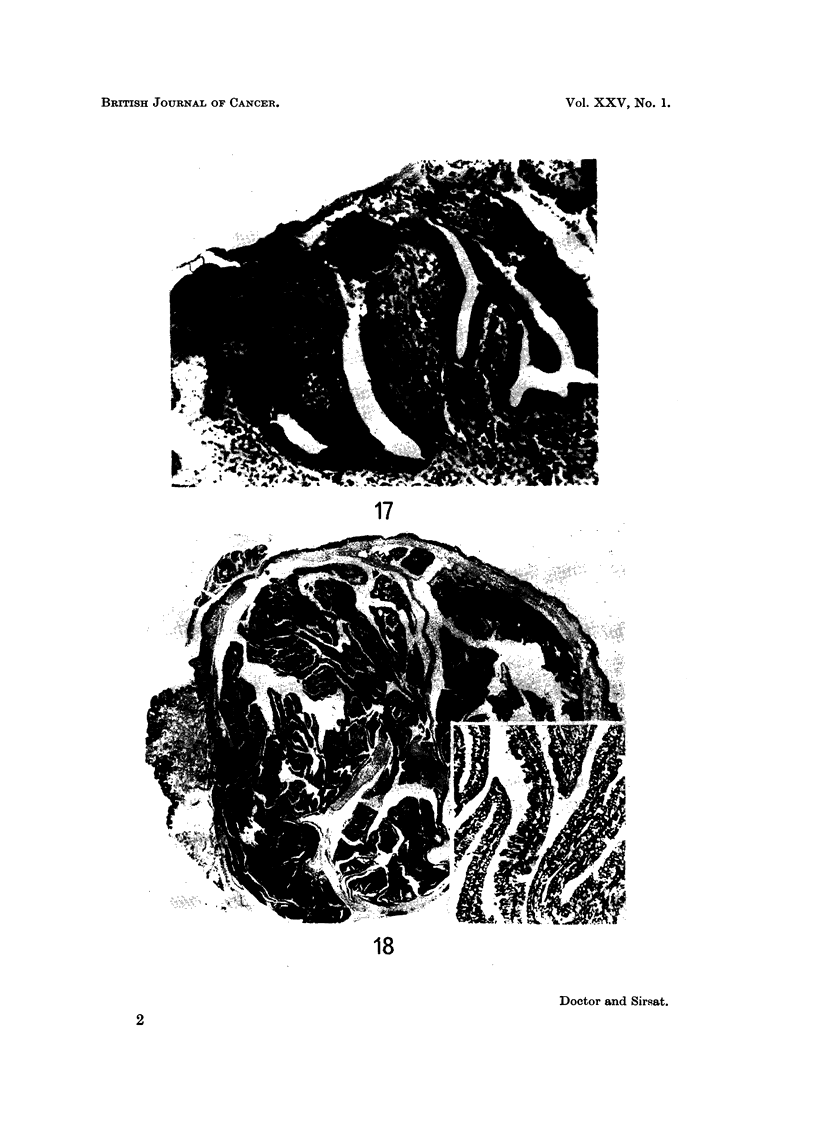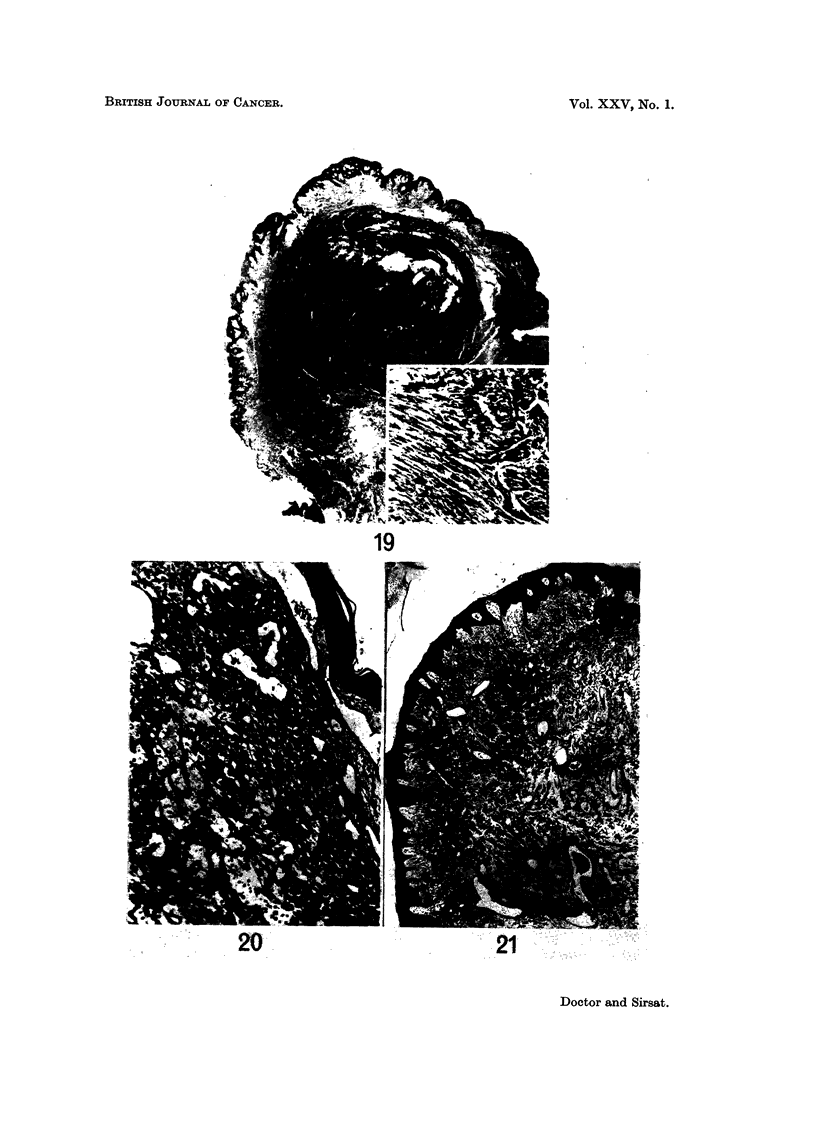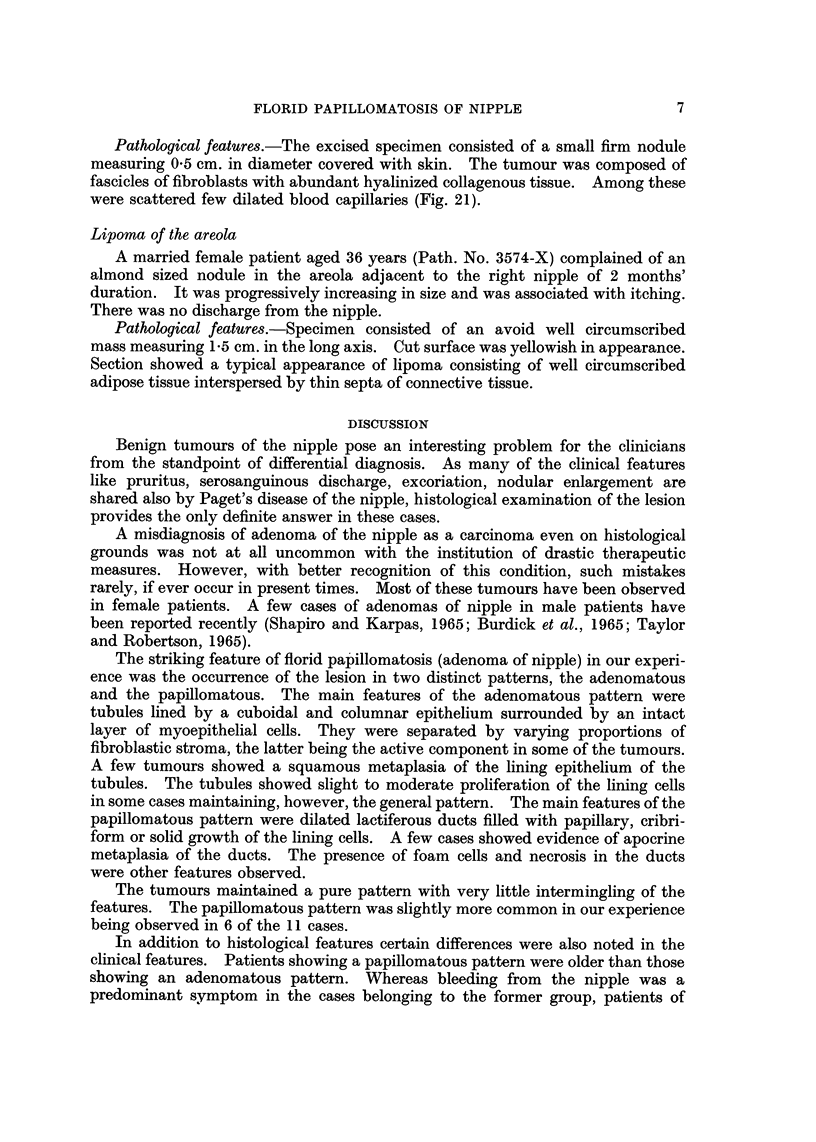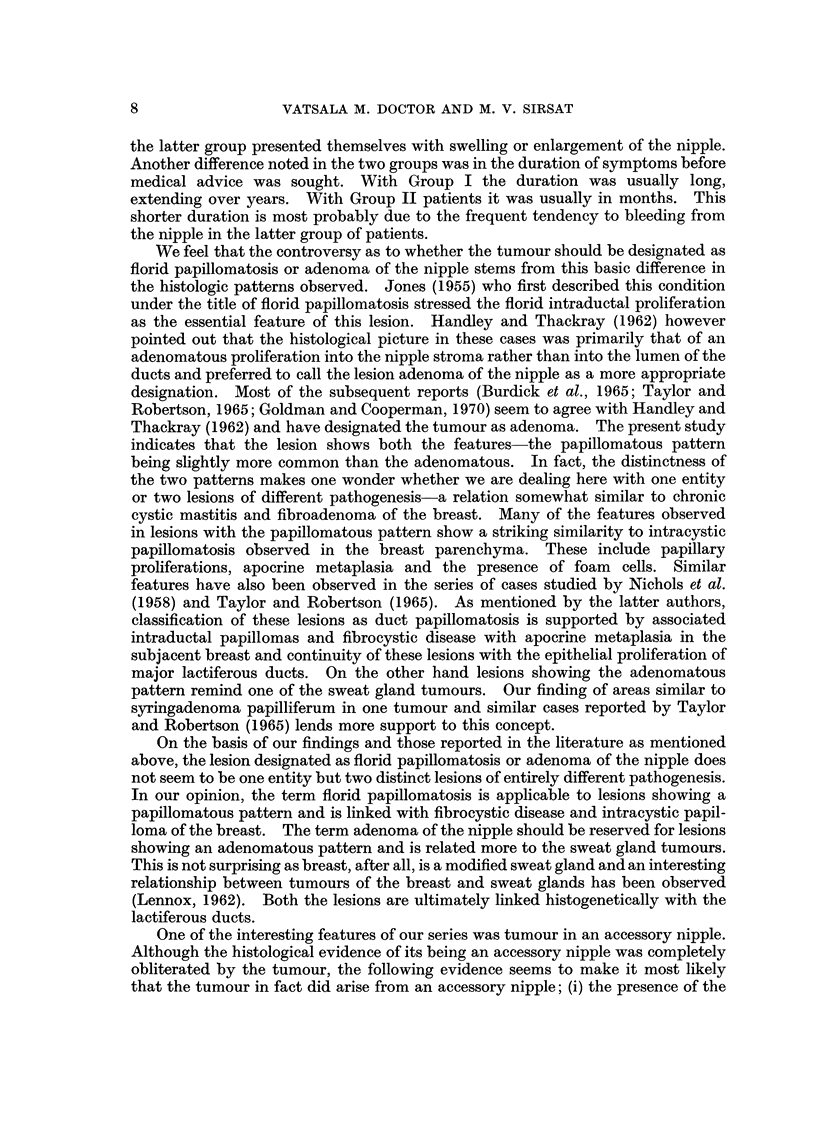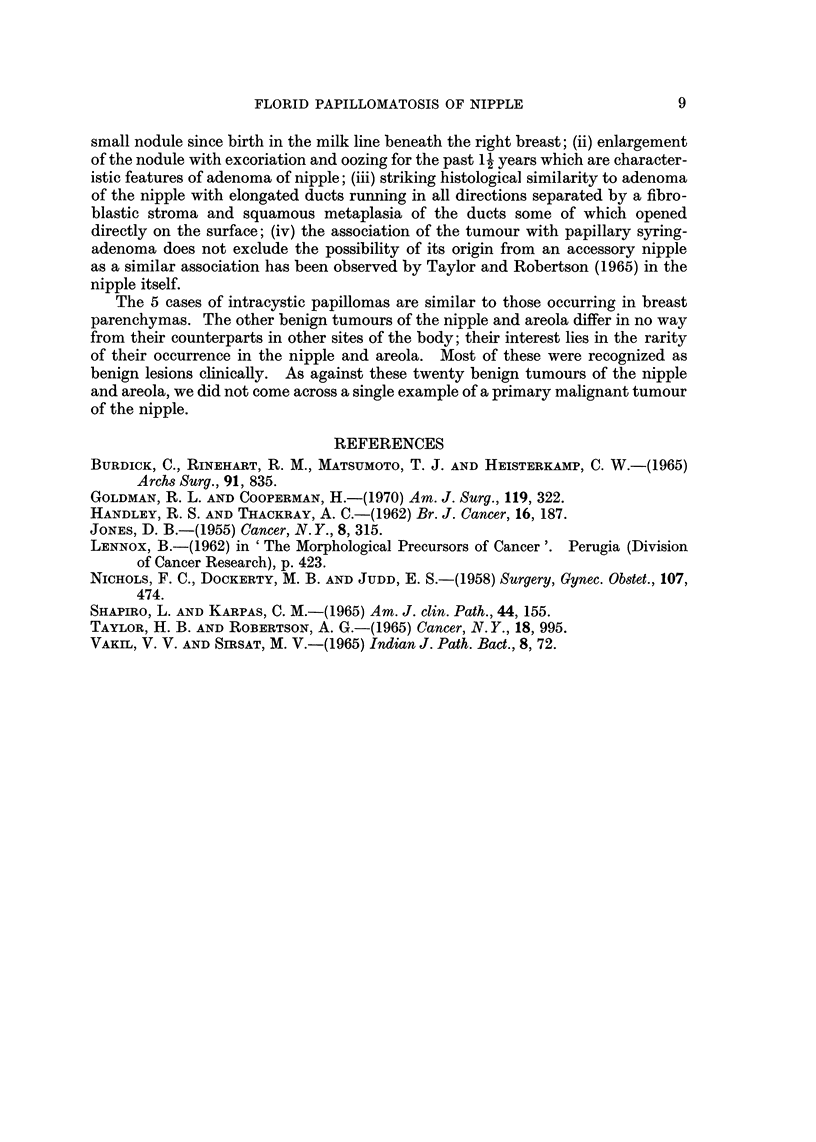# Florid Papillomatosis (Adenoma) and other Benign Tumours of the Nipple and Areola

**DOI:** 10.1038/bjc.1971.1

**Published:** 1971-03

**Authors:** Vatsala M. Doctor, M. V. Sirsat

## Abstract

**Images:**


### BRITISH JOURNAL OF CANCER

VOL. XXV MARCH, 1971 NO. 1

### FLORID PAPILLOMATOSIS (ADENOMA) AND OTHER BENIGN TUMOURS OF THE NIPPLE AND AREOLA

VATSALA M. DOCTOR AND M. V. SIRSAT

*From the Department of Pathology, Tata Memorial Hospital, Bombay, India*

Received for publication December 14, 1970

**SUMMARY.—The paper deals with 20 benign tumours of the nipple and areola. The most common lesion was adenoma or florid papillomatosis (11 cases). On the basis of clinical and histological differences, the cases were divided into two groups.**

**Group I.—Tumours showing an adenomatous pattern (5 cases) appropriately termed adenoma of the nipple. Chief clinical features were younger age, long duration and enlargement of the nipple as a predominant symptom. Histological characteristics were elongated tubules separated by a varying amount of fibrous stroma and squamous metaplasia in some of the cases.**

**Group II.—Tumours showing a papillomatous pattern (6 cases) appropriately designated as florid papillomatosis. Clinically, the patients were older. Chief complaint was bleeding from the nipple of a few months' duration. Histologically, dilated lactiferous ducts were seen filled with papillary, cribriform and solid growth of cells. Other features such as apocrine metaplasia, foam cells and central necrosis were observed.**

**The interpretation of this grouping is discussed.**

**A case of adenoma of an accessory nipple is reported.**

**Other benign tumours observed were five papillomas, one leiomyoma, one haemangioma, one fibroma and one lipoma.**

As against the extremely common involvement of the mammary gland by neoplastic processes, primary tumours of the nipple are indeed rare. The commonest among them is the florid papillomatosis or adenoma of the nipple, an entity first described by Jones in 1955, which often presents itself with classical signs and symptoms of Paget's disease and is easily confused histologically with carcinoma.

TABLE I.—*Benign Tumours of the Nipple and Areola*

| | Number of cases |
|---|---|
| Florid papillomatosis (adenoma) | 11 |
| Intracystic papilloma | 5 |
| Leiomyoma | 1 |
| Haemangioma | 1 |
| Fibroma | 1 |
| Lipoma | 1 |
| Total | 20 |

This presentation consists of 20 cases of benign tumours of the nipple and areola encountered at the Tata Memorial Hospital, during the past 30 years. Table I shows the break-up of these 20 cases.

### FLORID PAPILLOMATOSIS (ADENOMA OF THE NIPPLE)

On the basis of certain clinical and histological differences observed, the patients were divided into two groups.

Group I.—Those showing an adenomatous pattern (5 cases).

Group II.—Those showing a papillomatous pattern (6 cases).

### *Clinical Features*

*Group I.*—Table II summarizes the clinical features of cases showing an adenomatous pattern.

TABLE II.—*Summary of Clinical Data on Cases Showing an Adenomatous Pattern*

| Serial No. | Path. No. | Age of onset (years) | Sex | Community | Chief complaints | Duration |
|---|---|---|---|---|---|---|
| 1* | C-2 | 30 | Female | Not mentioned | Enlargement of the right nipple with pain | 8 months |
| 2 | 6518-B | 40 | Female | Moslem | Continuous pain right nipple and areola associated with serous discharge. Nipple fixed and retracted | 5 years |
| 3 | 3816-F | 36 | Female | Hindu | Swelling left nipple—gradually increasing in size | 5 years |
| 4 | 10598-AA | 24 | Female | Hindu | Swelling beneath the right breast since birth. Recent history of enlargement, ulceration with serous discharge | 1½ years |
| 5 | 1652-AB | 23 | Female | Hindu | Swelling right nipple | 1 year |

* This case is reported in detail by Vakil and Sirsat (1965).

TABLE III.—*Summary of Clinical Data on Cases Showing Papillomatous Pattern*

| Serial No. | Path. No. | Age of onset (years) | Sex | Community | Chief complaints | Duration |
|---|---|---|---|---|---|---|
| 1 | 6157-B | 60 | Female | Moslem | Irritation of the right nipple with bleeding | 4 months |
| 2 | 8710-C | 40 | Female | Hindu | Excoriation and irritation of the left nipple with bloodstained discharge | 8 months |
| 3 | 1824-F | 39 | Female | Hindu | Hard papillary growth left nipple | 2 months |
| 4 | 76-AB | 30 | Female | Hindu | Bleeding from the right nipple with " eczema " | 10 years |
| 5 | 2966-AB | 32 | Female | Hindu | Bleeding from the right nipple | 4 months |
| 6 | 3156-AB | 47 | Female | Hindu | Bleeding from the right nipple with nodular swelling | 1 year |

It can be seen that the patients belonged to the third and the fourth decades. The predominant presenting symptom was swelling of the nipple, in 2 cases associated with pain and serous discharge. There was no history of bloodstained discharge in a single case.

*Group II.*—Table III summarizes clinical features of cases showing a papillomatous pattern.

The patients showing a papillomatous pattern were women in their fourth decade of life or later. Bleeding from the nipple was the presenting symptom in 5 out of 6 cases. It was associated with excoriation and swelling in two cases each.

### *Pathological Features*

### *Group I*

*Gross observations.*—The nipple was enlarged varying in size from 0·8 to 4 cm. in diameter. On cutting, the entire nipple was occupied by a firm, greyish white nodule with ill-defined margins.

*Microscopic features.*—The tumours showed elongated tubules running in various directions separated by varying amounts of fibroblastic stroma (Fig. 1, 2 and 3). The tubules were lined by a regular double layer of epithelium, an inner columnar or cuboidal and an outer elongated myoepithelial. At places, the tubules showed slight to moderate proliferation of the lining epithelium. The nuclei were regular and uniform. One tumour showed extensive squamous metaplasia of the tubules (Fig. 3 and 4). The surface epithelium covering the tumour was intact in most of the cases with tubules opening on to it. Superficially, the tubules were dilated and showed squamous metaplasia, their lumina being filled with keratinized material (Fig. 2). The latter showed at places evidence of calcification. The tumour in the accessory nipple showed mainly an adenomatous pattern with areas similar to syringadenoma papilliferum of the skin.

### *Group II*

*Gross observations.*—The specimens showed a firm enlarged nipple measuring up to 1·5 cm. in diameter. The surface was irregular and excoriated. The cut surface showed the nipple replaced by a whitish homogenous mass.

*Microscopic features.*—The chief feature of this pattern was the presence of large lactiferous ducts showing marked intraductal proliferation of the lining cells in the form of papillary, cribriform or solid growth (Fig. 5, 6, 7 and 8). They were surrounded by a single layer of myoepithelial cells. The cells were cuboidal or polygonal and showed ovoid uniform nuclei. Two tumours showed apocrine metaplasia of the lining cells (Fig. 9). Other features of this pattern were the presence of foam cells (Fig. 10), and necrosis in the centre of the ducts (Fig. 11).

The adjoining breast parenchyma, whenever available for study, showed evidence of chronic cystic mastitis with apocrine metaplasia. A large intracystic papilloma was seen in two cases (Fig. 12).

The tumours could be very easily separated into one of the two types as there was very little overlap of the characteristic features mentioned above, *i.e.* papillary areas, apocrine metaplasia and presence of foam cells were seen in none of the tumours showing an adenomatous pattern. Similarly, tubular arrangement and squamous metaplasia of an appreciable degree were not seen in tumours showing a papillomatous pattern. Table IV summarizes the main features of the two patterns.

One tumour was observed in an accessory nipple. Being the first of its kind observed in this unusual site, a detailed account of the clinical and pathological features is given below.

TABLE IV.—*Summary of Clinical and Histological Features of Tumours Showing Adenomatous and Papillomatous Patterns*

| | Adenomatous pattern | Papillomatous pattern |
|---|---|---|
| Clinical features: | . Patients in 3rd and 4th decades | . Patients in 4th decade and over |
| | Long duration extending over years | Short duration usually in months |
| | Presenting symptom—swelling or enlargement of the nipple | Presenting symptom bleeding from nipple |
| Histological features: | . Tubular arrangement | . Intraductal papillary, cribriform and solid growth of cells |
| | Squamous metaplasia of the tubules | Apocrine metaplasia of the ducts |
| | Fibroblastic stroma | Presence of foam cells and central necrosis in the ducts |
| | Resemblance to sweat gland tumours | Resemblance to intracystic papillomatosis of breast parenchyma |

### *Case History*

A female patient aged 26 years (Path. No. 10598-AA) complained of a small nodule on the lateral aspect of the chest beneath the right breast since birth. For the past $1\frac{1}{2}$ years, the nodule had gradually enlarged in size with ulceration and serous discharge. One examination, there was an ulcerated brownish papillary growth over the skin beneath the right breast. The lesions was completely excised with a margin of normal skin surrounding the tumour.

The specimen received in the laboratory consisted of a brownish warty mass measuring 4 × 2 cm. in size surrounded by about 1 cm. of normal skin. On cutting, the tumour was well circumscribed and presented a greyish-white appearance.

On histological examination, the entire epidermis covering the tumour was replaced by a double layer of epithelium arranged in a papillary fashion and as crypts dipping into the dermis (Fig. 13). The stroma of the papillae and the crypts showed dense collections of lymphocytes and plasma cells. The appearance was reminiscent of syringadenoma papilliferum of the skin (Fig. 14). In the deeper portion were seen elongated tubules running in various directions (Fig. 15). This portion of the tumour occupied the entire dermis reaching up to the subcutaneous tissue. The cells lining the tubules were mainly cuboidal or columnar surrounded by a single layer of flattened cells. At places the tubules showed a moderate degree of proliferation of the lining cells. Some of the tubules showed squamous metaplasia of the lining epithelium (Fig. 16). Superficially, the tubules were running perpendicular to the surface and opening directly on to it (Fig. 17). The deeper portion of the tumour showed a structure indistinguishable from that observed in adenoma of the nipple showing an adenomatous pattern.

### OTHER BENIGN TUMOURS OF THE NIPPLE AND AREOLA

### *Intracystic papilloma of the nipple*

There were 5 cases of intracystic papilloma of the nipple. Table V summarizes the clinical features in these cases.

TABLE V.—*Clinical Data on 5 Cases of Intracystic Papilloma of Nipple*

| Serial No. | Path. No. | Sex | Age (years) | Chief complaints | Clinical findings |
|---|---|---|---|---|---|
| 1 | B-608 | Female | 51 | Serosanguinous discharge from nipple for 1 year | Nipple " eczematous ". A cord-like mass measuring 4 cm. in the long axis involving the nipple and adjoining areola |
| 2 | 856-J | Female | 44 | Bleeding from left nipple for 20 days | The left nipple retracted, ulcerated and shows a papillomatous growth |
| 3 | 534-P | Female | 65 | Serosanguinous discharge from right nipple of 6 month's duration | The nipple enlarged 4 times the normal size |
| 4 | 2306-U | Female | 38 | History not available | — |
| 5 | 514-Z | Female | 29 | Serosanguinous discharge | A firm nodule felt in the nipple |

*Pathological features.*—Gross examination of the excised specimens revealed enlarged nipples which were also retracted and, in one case, ulcerated. On cutting, dilated cystic ducts filled with whitish papillary masses were seen.

Microscopic examination showed dilated and cystic ducts just beneath the nipple epithelium. They were partially or completely filled with a papillary mass composed of delicate anastomosing papillary processes covered by a columnar epithelium (Fig. 18). The cells showed ovoid, uniform nuclei. The appearance of the papillomas was indistinguishable from that observed in intracystic papillomas of the breast parenchyma.

### *Leiomyoma of the nipple*

A male patient aged 40 years (Path. No. 2230-X) complained of enlargement of the nipple of 5 years' duration. It was increasing in size for one year and had become painful. There was no discharge from the nipple.

*Pathological features.*—The stratified squamous epithelium was raised by a well circumscribed nodule composed of intersecting bundles of elongated fusiform cells. The nuclei were elongated and showed blunt rounded ends. The cytoplasm showed the presence of myofibrils (Fig. 19).

### *Haemangioma of the nipple*

A male patient aged 16 years (Path. No. 1086-Y) complained of bleeding from the nipple. On examination, a small nodular growth was observed involving the left nipple and adjoining areola.

*Pathological features.*—Just beneath the epidermis were seen proliferated capillary spaces lined by a single layer of swollen endothelial cells. The lumina were filled with red blood cells (Fig. 20).

*Fibroma of the nipple*

A female patient aged 30 years (Path. No. 7256-C) came with the complaints of a small warty growth in the left nipple of 2 months' duration. It was accompanied by ulceration and bloodstained discharge for 8 days. Menstrual and obstetric history revealed nothing abnormal. On examination, there was a small pedunculated papillomatous growth involving the left nipple with an excoriated surface. It exuded serous fluid on pressure.

---

EXPLANATION OF PLATES

FIG. 1.—Photomicrograph showing typical adenomatous pattern consisting of tubules running in various directions separated by fibroblastic stroma. H. and E. × 22. (Path. No. 6518-B).

FIG. 2.—Photomicrograph shows tubules arranged in an adenomatous pattern. Just beneath the epidermis are seen dilated tubules filled with keratinized material. H. and E. × 13. (Path. No. 3816-F).

FIG. 3.—Photomicrograph of a tumour showing extensive squamous metaplasia of the tubules which are converted into solid cords. H. and E. × 78. (Path. No. C-2).

FIG. 4.—High power view of the same tumour shown in Fig. 3. H. and E. × 110. (Path. No. C-2).

FIG. 5.—Photomicrograph of entire tumour showing papillomatous pattern. Inset shows dilated ducts filled with solid growth of cells. H. and E. × 7; Inset × 65). (Path. No. 76-AB).

FIG. 6.—Photomicrograph of tumour showing papillary, solid and cribriform patterns. H. and E. × 58. (Path. No. 2966-AB).

FIG. 7.—Photomicrograph showing two dilated tubules completely filled with proliferated cells—papillomatous pattern. H. and E. × 58. (Path. No. 76-AB).

FIG. 8.—A tumour showing papillomatous pattern. Note the dilated lactiferous ducts partially or completely filled with proliferated cells. H. and E. × 16. (Path. No. 3156-AB).

FIG. 9.—Photomicrograph shows ducts showing an apocrine metaplasia of the lining epithelium —same case as shown in Fig. 5. H. and E. × 70. (Path. No. 76-AB).

FIG. 10.—Photomicrograph shows a dilated duct filled with foam cells. H. and E. × 70. (Path. No. 76-AB).

FIG. 11.—Photomicrograph shows the centre of a duct filled with necrotic material. H. and E. × 61. (Path. No. 2966-AB).

FIG. 12.—Photomicrograph shows an intracystic papilloma adjoining a nipple showing florid papillomatosis. Areas similar to the papilloma can be seen in the upper part of the lesion. H. and E. × 4. (Path. No. 76-AB).

FIG. 13.—Photomicrograph of a tumour in an accessory nipple. The entire surface is replaced by tumour cells arranged in a manner reminiscent of syringadenoma papilliferum of skin. The deeper portion of the tumour is composed of tubules arranged in an adenomatous pattern. H. and E. × 4. (Path. No. 10598-AA).

FIG. 14.—High power view of the superficial part of the tumour shown in Fig. 13. It shows epithelium thrown into papillary folds with collections of lymphocytes and plasma cells in the stroma underneath. The appearance is indistinguishable from syringadenoma papilliferum of the skin. H. and E. × 60. (Path. No. 10598-AA).

FIG. 15.—High power of the deeper portion of the tumour shown in Fig. 13, composed of elongated tubules running in various directions. H. and E. × 25. (Path. No. 10598-AA).

FIG. 16.—Same case as Fig. 13. A tubule shows squamous metaplasia of the lining epithelium. H. and E. × 230. (Path. No. 10598-AA).

FIG. 17.—Same case as Fig. 13. Tubules are seen opening on the surface. H. and E. × 84. (Path. No. 10598-AA).

FIG. 18.—Photomicrograph showing a papilloma of the nipple. H. and E. × 7½; Inset × 84. (Path. No. 534-P).

FIG. 19.—Photomicrograph of a leiomyoma of the nipple. Inset shows high power view of the tumour cells. H. and E. × 11; Inset × 80. (Path. No. 2230-X).

FIG. 20.—Photomicrograph shows histological appearance of haemangioma of the nipple. H. and E. × 80. (Path. No. 1086-Y).

FIG. 21.—Photomicrograph of a fibroma of the nipple composed of loosely arranged fibroblasts separated by abundant collagenous fibres and scattered capillaries. H. and E. × 12. (Path. No. 7256-C).

1

3

2

4

6

5

Doctor and Sirsat.

7

8

9

10

11

12

13

14

15

16

17

18

Doctor and Sirsat.

19

20

21

*Pathological features.*—The excised specimen consisted of a small firm nodule measuring 0·5 cm. in diameter covered with skin. The tumour was composed of fascicles of fibroblasts with abundant hyalinized collagenous tissue. Among these were scattered few dilated blood capillaries (Fig. 21).

*Lipoma of the areola*

A married female patient aged 36 years (Path. No. 3574-X) complained of an almond sized nodule in the areola adjacent to the right nipple of 2 months' duration. It was progressively increasing in size and was associated with itching. There was no discharge from the nipple.

*Pathological features.*—Specimen consisted of an avoid well circumscribed mass measuring 1·5 cm. in the long axis. Cut surface was yellowish in appearance. Section showed a typical appearance of lipoma consisting of well circumscribed adipose tissue interspersed by thin septa of connective tissue.

### DISCUSSION

Benign tumours of the nipple pose an interesting problem for the clinicians from the standpoint of differential diagnosis. As many of the clinical features like pruritus, serosanguinous discharge, excoriation, nodular enlargement are shared also by Paget's disease of the nipple, histological examination of the lesion provides the only definite answer in these cases.

A misdiagnosis of adenoma of the nipple as a carcinoma even on histological grounds was not at all uncommon with the institution of drastic therapeutic measures. However, with better recognition of this condition, such mistakes rarely, if ever occur in present times. Most of these tumours have been observed in female patients. A few cases of adenomas of nipple in male patients have been reported recently (Shapiro and Karpas, 1965; Burdick *et al.*, 1965; Taylor and Robertson, 1965).

The striking feature of florid papillomatosis (adenoma of nipple) in our experience was the occurrence of the lesion in two distinct patterns, the adenomatous and the papillomatous. The main features of the adenomatous pattern were tubules lined by a cuboidal and columnar epithelium surrounded by an intact layer of myoepithelial cells. They were separated by varying proportions of fibroblastic stroma, the latter being the active component in some of the tumours. A few tumours showed a squamous metaplasia of the lining epithelium of the tubules. The tubules showed slight to moderate proliferation of the lining cells in some cases maintaining, however, the general pattern. The main features of the papillomatous pattern were dilated lactiferous ducts filled with papillary, cribriform or solid growth of the lining cells. A few cases showed evidence of apocrine metaplasia of the ducts. The presence of foam cells and necrosis in the ducts were other features observed.

The tumours maintained a pure pattern with very little intermingling of the features. The papillomatous pattern was slightly more common in our experience being observed in 6 of the 11 cases.

In addition to histological features certain differences were also noted in the clinical features. Patients showing a papillomatous pattern were older than those showing an adenomatous pattern. Whereas bleeding from the nipple was a predominant symptom in the cases belonging to the former group, patients of the latter group presented themselves with swelling or enlargement of the nipple. Another difference noted in the two groups was in the duration of symptoms before medical advice was sought. With Group I the duration was usually long, extending over years. With Group II patients it was usually in months. This shorter duration is most probably due to the frequent tendency to bleeding from the nipple in the latter group of patients.

We feel that the controversy as to whether the tumour should be designated as florid papillomatosis or adenoma of the nipple stems from this basic difference in the histologic patterns observed. Jones (1955) who first described this condition under the title of florid papillomatosis stressed the florid intraductal proliferation as the essential feature of this lesion. Handley and Thackray (1962) however pointed out that the histological picture in these cases was primarily that of an adenomatous proliferation into the nipple stroma rather than into the lumen of the ducts and preferred to call the lesion adenoma of the nipple as a more appropriate designation. Most of the subsequent reports (Burdick *et al.*, 1965; Taylor and Robertson, 1965; Goldman and Cooperman, 1970) seem to agree with Handley and Thackray (1962) and have designated the tumour as adenoma. The present study indicates that the lesion shows both the features—the papillomatous pattern being slightly more common than the adenomatous. In fact, the distinctness of the two patterns makes one wonder whether we are dealing here with one entity or two lesions of different pathogenesis—a relation somewhat similar to chronic cystic mastitis and fibroadenoma of the breast. Many of the features observed in lesions with the papillomatous pattern show a striking similarity to intracystic papillomatosis observed in the breast parenchyma. These include papillary proliferations, apocrine metaplasia and the presence of foam cells. Similar features have also been observed in the series of cases studied by Nichols *et al.* (1958) and Taylor and Robertson (1965). As mentioned by the latter authors, classification of these lesions as duct papillomatosis is supported by associated intraductal papillomas and fibrocystic disease with apocrine metaplasia in the subjacent breast and continuity of these lesions with the epithelial proliferation of major lactiferous ducts. On the other hand lesions showing the adenomatous pattern remind one of the sweat gland tumours. Our finding of areas similar to syringadenoma papilliferum in one tumour and similar cases reported by Taylor and Robertson (1965) lends more support to this concept.

On the basis of our findings and those reported in the literature as mentioned above, the lesion designated as florid papillomatosis or adenoma of the nipple does not seem to be one entity but two distinct lesions of entirely different pathogenesis. In our opinion, the term florid papillomatosis is applicable to lesions showing a papillomatous pattern and is linked with fibrocystic disease and intracystic papilloma of the breast. The term adenoma of the nipple should be reserved for lesions showing an adenomatous pattern and is related more to the sweat gland tumours. This is not surprising as breast, after all, is a modified sweat gland and an interesting relationship between tumours of the breast and sweat glands has been observed (Lennox, 1962). Both the lesions are ultimately linked histogenetically with the lactiferous ducts.

One of the interesting features of our series was tumour in an accessory nipple. Although the histological evidence of its being an accessory nipple was completely obliterated by the tumour, the following evidence seems to make it most likely that the tumour in fact did arise from an accessory nipple; (i) the presence of the small nodule since birth in the milk line beneath the right breast; (ii) enlargement of the nodule with excoriation and oozing for the past $1\frac{1}{2}$ years which are characteristic features of adenoma of nipple; (iii) striking histological similarity to adenoma of the nipple with elongated ducts running in all directions separated by a fibroblastic stroma and squamous metaplasia of the ducts some of which opened directly on the surface; (iv) the association of the tumour with papillary syringadenoma does not exclude the possibility of its origin from an accessory nipple as a similar association has been observed by Taylor and Robertson (1965) in the nipple itself.

The 5 cases of intracystic papillomas are similar to those occurring in breast parenchymas. The other benign tumours of the nipple and areola differ in no way from their counterparts in other sites of the body; their interest lies in the rarity of their occurrence in the nipple and areola. Most of these were recognized as benign lesions clinically. As against these twenty benign tumours of the nipple and areola, we did not come across a single example of a primary malignant tumour of the nipple.

### REFERENCES

BURDICK, C., RINEHART, R. M., MATSUMOTO, T. J. AND HEISTERKAMP, C. W.—(1965) *Archs Surg.*, **91**, 835.
GOLDMAN, R. L. AND COOPERMAN, H.—(1970) *Am. J. Surg.*, **119**, 322.
HANDLEY, R. S. AND THACKRAY, A. C.—(1962) *Br. J. Cancer*, **16**, 187.
JONES, D. B.—(1955) *Cancer, N.Y.*, **8**, 315.
LENNOX, B.—(1962) in ‘ The Morphological Precursors of Cancer ’. Perugia (Division of Cancer Research), p. 423.
NICHOLS, F. C., DOCKERTY, M. B. AND JUDD, E. S.—(1958) *Surgery, Gynec. Obstet.*, **107**, 474.
SHAPIRO, L. AND KARPAS, C. M.—(1965) *Am. J. clin. Path.*, **44**, 155.
TAYLOR, H. B. AND ROBERTSON, A. G.—(1965) *Cancer, N.Y.*, **18**, 995.
VAKIL, V. V. AND SIRSAT, M. V.—(1965) *Indian J. Path. Bact.*, **8**, 72.